# Outcomes of a Telephone-Based Frailty and Functional Assessment

**DOI:** 10.1093/geroni/igab046.3003

**Published:** 2021-12-17

**Authors:** Stephanie Denise Sison, Karla Tejada Arias, Natalie Newmeyer, Racheli Schoenburg, Carolina Fonseca Valencia, Dae Kim

**Affiliations:** 1 Beth Israel Deaconess Medical Center / VA Boston New England GRECC, Boston, Massachusetts, United States; 2 BIDMC, Boston, Massachusetts, United States; 3 Marcus Institute for Aging Research, Boston, Massachusetts, United States; 4 BIDMC, Providence, Rhode Island, United States; 5 Hebrew SeniorLife, Boston, Massachusetts, United States

## Abstract

With the goal of increasing the clinical use of frailty, we piloted a quality improvement project to determine the feasibility and utility of a telephone-based frailty and functional assessment. We identified 122 established patients with serious medical illness from an academic geriatrics clinic. A geriatric fellow assessed the functional status and conducted the Mini Nutritional Assessment, telephone-MoCA, and Geriatric Depression Scale to generate a deficit-accumulation frailty index (FI) score which was automatically calculated through the electronic medical record. A note was then generated to inform the providers of the details of the assessment and to provide recommendations based on the findings. From November 2020 to March 2021, 104 out of 122 (85.2%) established patients (mean [SD]: 83.4 [7.1], 66% female, 81% White, and mean [SD] FI: 0.32 [0.17]) agreed and proceeded with the assessment. One month after the call, we found that the assessment was included in the clinical decision-making of 55 out of 100 patients seen by their primary care provider. The top 3 incorporated recommendations were chronic disease management based on frailty status (n=56), lifestyle change and counseling to prevent frailty progression (n=44), and management of cognition and mood (n=18). Management of physical status including referral to PT and OT were incorporated in 15 encounters. Our results suggest that a telephone-based frailty and functional assessment is feasible and has value-added contributions in improving the care of older adults through providing a holistic view of their health status.

